# Mother-in-law childcare and perinatal depression in rural Pakistan

**DOI:** 10.1177/17455057221141288

**Published:** 2022-12-05

**Authors:** Esther O Chung, Ashley Hagaman, Amina Bibi, Allison Frost, Sarah C Haight, Siham Sikander, Joanna Maselko

**Affiliations:** 1 Epidemiology, University of North Carolina at Chapel Hill, Chapel Hill, NC, USA; 2 Carolina Population Center, University of North Carolina at Chapel Hill, Chapel Hill, NC, USA; 3 Department of Social and Behavioral Sciences, Yale School of Public Health, New Haven, CT, USA; 4 Center for Methods in Implementation and Prevention Science, Yale School of Public Health, New Haven, CT, USA; 5 Global Institute of Human Development, Shifa Tameer-e-Millat University, Islamabad, Pakistan; 6 Human Development Research Foundation, Islamabad, Pakistan; 7 Department of Primary Care and Mental Health, University of Liverpool, Liverpool, UK

**Keywords:** child care, family conflict, intergenerational relations, maternal mental health, mother-in-law, Pakistan, social support, perinatal depression

## Abstract

**Background:**

Mothers-in-law often provide key childcare support to daughters-in-law during the perinatal period that may enhance maternal mental health. Yet, poor mother-in-law/daughter-in-law relationships may be associated with maternal depression. The extent to which mother-in-law childcare involvement affects perinatal depression may differ across contexts of family conflict.

**Objective:**

We explored the relationship between mother-in-law childcare and daughter-in-law perinatal depression in rural Pakistan across contexts of family conflict.

**Methods:**

Data on 783 women came from the Bachpan Cohort, a birth cohort in Pakistan. Maternally-reported mother-in-law childcare was assessed at 3 and 12 months postpartum using a 24-h recall and categorized into no, low, and high involvement. Major depression was captured at 3 and 12 months using the Structured Clinical Interview for Diagnostic and Statistical Manual of Mental Disorders IV. Family conflict was captured using three items from the Life Events Checklist. Log-Poisson models were used to estimate cross-sectional associations between mother-in-law childcare and perinatal depression, stratified by family conflict.

**Results:**

Mother-in-law childcare was common in the first year postpartum. The association between mother-in-law childcare and perinatal depression differed by the presence of family conflict and postpartum timing. At 3 months postpartum, low and high mother-in-law childcare (vs no involvement) were associated with a lower prevalence of depression regardless of family conflict. At 12 months postpartum, among families with no conflict, low mother-in-law childcare (vs no involvement) was associated with lower perinatal depression; however, among families with conflict, high mother-in-law childcare was associated with increased perinatal depression.

**Conclusion:**

Our findings highlight the complexities of associations between mother-in-law childcare support and perinatal depression in the first year after birth. Mother-in-law childcare in the immediate postpartum period was beneficial for mothers. Understanding the source, amount, timing, and context of social support is necessary to inform research and interventions that aim to improve maternal mental health.

## Introduction

Mental health during the perinatal period, defined as the time from pregnancy to 1 year postpartum, has ramifications for a mother’s psychosocial well-being, physical health, and quality of life throughout the life course.^1,2^ The sequelae may extend to children, as perinatal mental health conditions may increase the risk of negative birth outcomes, poor nutrition, and suboptimal physical, cognitive, and socioemotional developmental trajectories.^3,4^ High-quality perceived social support may provide notable prevention for depression well after the perinatal period.^5,6^ Even in situations of adverse environments where persistent poverty, domestic violence, and access to limited resources, exposure to positive social and instrumental support may prevent and alleviate depression.^7,8^ However, depression may directly impact a mother’s ability to maintain vital social support, and exposure to close relationships that have little support and chronic conflict may exacerbate depression symptoms.

The type, timing, source, and context of social support may affect its relationship with perinatal depression. Traditional practices surrounding childbirth and increased postpartum support are protective against perinatal depression.^9^ Such practices include supportive rest, increased care, provision of special foods, and relief from household chores in the months after childbirth.^10^ In Pakistan, this practice is called *chilla*, and research from our cohort found that *chilla* reduced maternal depressive symptoms and the prevalence of major depressive disorder.^5^ Outside of the immediate postpartum period, our past analyses found that perceived emotional support may be more meaningful than instrumental support.^6^ Other studies found that it was the number of individuals or the specific individual (e.g. husband, in-law) who delivered the support that impacted depressive outcomes.^11–13^

While prior literature has examined the impacts of paternal support on maternal psychosocial outcomes,^8,14–18^ little attention has focused on other core family members, particularly those rooted in joint families. In South Asia, patrilocal tradition supports a married woman joining her husband’s household, often including cohabiting in-laws. The mother-in-law (MIL)/daughter-in-law (DIL) relationship may be deeply entwined, where child-rearing, housework, and family support are shared tasks.^19^ Prior work in our cohort demonstrated a high percentage of MIL providing childcare, which supported child growth, and cognitive and socioemotional development.^20^ Yet, there is limited literature on how MIL childcare involvement affects the mother’s broader mental health. MIL involvement in childcare could be a welcome source of support to mothers, ultimately decreasing the risk of perinatal depression. Alternatively, MIL involvement may cause stress and increase the risk of depression in mothers in the postpartum period, as MIL and DIL navigate day-to-day childcare and child-rearing decisions.

Ethnographic studies in India, Nepal, and China highlight the complexity of the MIL/DIL relationship, particularly noting tension, power struggle, and conflict.^19,21–23^ Qualitative work among pregnant women at risk for anxiety in Pakistan found that women’s greatest source of worry and stress during pregnancy was their MIL.^24^ In particular, women faced difficulty exercising self-advocacy related to the burden of household chores and reproductive health decisions in fear of upsetting their husbands, MILs, and sisters-in-law. Moreover, MILs have strong influences on DILs’ healthcare access and behaviors. Ethnographic work in Pakistan found that MILs have vested authority and decision-making power on pregnancy-related issues and the MIL/DIL relationship quality can dictate whether the DIL receives healthcare.^25^ Such relationship dynamics may lead to increased conflict, stress, and adverse outcomes for mothers. Research has found that poor MIL/DIL relationships were associated with increased maternal depression.^26–28^ A study in rural China found that one-third of women who attempted suicide reported MIL conflict.^29^

In Pakistan and other patrilineal cultures, child-rearing is communal, with multiple family members providing care, especially female relatives such as MIL, aunts, and older sisters.^30^ MILs may be actively engaged in childcare in the first year of life and thus, play an important role in maternal depression, depending on the level of involvement and context. One hypothesis is that high levels of MIL involvement in childcare may help reduce maternal depression risk by providing greater social support. Another hypothesis is that for mothers with high self-efficacy or with more strained relations with MIL, little to no level of MIL childcare may be sufficient to benefit maternal mental health. Moreover, childcare support across the postpartum period changes over time as the child ages; MIL childcare in the first 3 months postpartum is qualitatively different than at 12 months. MIL support in childcare at 3 months may be more helpful for maternal mental health because this time period could be more stressful as mothers are still adjusting to the newborn, whereas MIL support at 12 months may be perceived as more intrusive because mothers may not require as much help. Exploring how MIL childcare at different time points in the postpartum period affects perinatal depression can help to inform when and how to intervene to improve maternal mental health.

Furthermore, beyond the MIL/DIL relationship, the broader family context may play an important role in the relationship between MIL childcare and perinatal depression. Specifically, general family conflict, such as arguments between family members (which may include members from the extended family that do not live nearby), tense/troubled relations with close relatives, and marital problems may moderate the impact of MIL childcare on perinatal depression. In settings with family conflict, greater childcare support from MIL may lead to tension, power imbalances, and feelings of inadequacy in DIL that could cause or worsen symptoms of depressive symptoms. Alternatively, MIL support in the absence of family conflict may simply reduce the burden of tasks, potentially alleviating stress or depressive symptoms. Examination of whether, how, and in what contexts MIL childcare across the postpartum period impacts perinatal depression may help to inform the potential timing and targets of social support interventions. Thus, in this study, we explore how MIL involvement in grandchild everyday care affects perinatal depression at 3 and 12 months postpartum across contexts of family conflict.

## Methods

### Study population

Data came from the Bachpan Cohort, a longitudinal birth cohort with an embedded cluster-randomized trial of a psychosocial maternal depression intervention in rural Pakistan. Details on the cohort are provided elsewhere.^31–33^ Briefly, pregnant women in the third trimester of pregnancy across 40 village clusters in the Kallar Syedan sub-district were recruited between 2014 and 2016. Women were eligible if they were 18 years or older, spoke Urdu, Punjabi, or Potohari, and intended to stay in the study area for at least 1 year. All eligible pregnant women were screened for depression using the Urdu-validated version of the Patient Health Questionnaire-9 (PHQ-9).^34^ For nearly every 3 depressed women (PHQ-9 ⩾ 10) enrolled in the trial, 1 non-depressed woman (PHQ-9 < 10) from the same village cluster was enrolled in the cohort, creating a population-representative sample with sampling weights. For this study, we used data from two cross-sectional waves: 3 and 12 months. Out of the 1154 women who were enrolled during the third trimester of pregnancy, 783 had available data on all key variables at 3 and 12 months postpartum.

### Measures

#### MIL childcare

MIL involvement in childcare was captured at 3 and 12 months postpartum using the Day-in-the-Life measure, a semi-structured interview in which mothers described the past 24 h from their child’s perspective^35^ (Appendix Table 1). The women reported household members’ participation in 11 child caregiving activities (e.g. feeding, playing, and caregiving support), and we isolated MIL involvement. Participation in 5 of the 11 items was asked about multiple times throughout the day (morning, afternoon, and evening): sleep routine, feeding, snacks (only afternoon and evening), changing diapers or clothes, and childcare when the mother is busy with chores. Consistent with our prior work,^20^ we only counted participation in each of these activities once to avoid overweighting these five activities. Then, we generated a total sum of the activities, ranging from 0 to 11. The summed score was heavily right-skewed; to help with fit and interpretability, we created a categorical score. After labeling zero values as no involvement, we selected the median value to define low and high involvement and create three categories: No (0), Low (1–5), and High (6 or higher) involvement.

#### Perinatal depression

Perinatal depression was assessed at baseline, 3, and 12 months using the current major depressive episode module of the Structured Clinical Interview for *Diagnostic and Statistical Manual of Mental Disorders: Fourth Edition* (*DSM*-IV) Disorders (SCID).^36^ The SCID has been culturally adapted across contexts for use among pregnant and postpartum women^37,38^ as well as translated to Urdu and previously used in Pakistan.^39^

#### Family conflict

We captured exposure to general family conflict at the third trimester of pregnancy (baseline) and 12 months using items from the Life Events Checklist. The Life Events Checklist is a modified version of the Life Events and Difficulties Schedule, a self-reported measure of potentially traumatic events and difficulties in the past year.^40^ It was culturally adapted to list the key set of events and difficulties seen in rural Pakistan, translated, tested, and previously used in the study area.^41,42^ We constructed a binary indicator for any endorsement of the following 3 items: (1) “You or other family members have had rows/quarrels among themselves,” (2) “Your relations with any of your close relatives or friends have been troubled,” and (3) “Your marital relations with your spouse have had problems.”

#### Covariates

Confounders of the exposure and outcome relationship included child gender and the following baseline characteristics: maternal age; maternal education (none, primary or middle school, secondary school or higher); parity (first pregnancy, 1–3 children, 4 or more children); whether a grandmother lives with the mother, household size, socioeconomic status (SES) (standardized asset index using a polychoric principal components analysis);^7,43^ and trial arm (control, intervention, non-depressed). Other baseline covariates utilized in weighting procedures included paternal education (none, primary to middle school, secondary school or higher) and any maternal experience of physical, psychological, or sexual intimate partner violence (IPV) in the past 12 months (measured with the World Health Organization Violence Against Women instrument).^44^

#### Statistical analysis

To estimate the associations between MIL childcare and perinatal depression, we conducted weighted generalized linear models with robust standard errors stratified by family conflict. To estimate prevalence ratios (PRs) and 95% confidence intervals (CI), we conducted log-binomial models. However, our models did not converge; therefore, we conducted log-Poisson models, which have been previously shown to be an alternative method.^45^ All models controlled for the following confounders, which were selected *a priori*: maternal age, maternal education, parity, child gender, grandmother living in the household, people per room, nuclear family, SES assets, and trial arm. Three-month models were stratified by baseline family conflict and adjusted for baseline outcomes. Twelve-month models were stratified by 12-month family conflict and adjusted for baseline and 3-month outcomes as well as 3-month MIL childcare.

To account for potentially informative loss to follow-up, we used stabilized inverse probability of censoring weights (IPCW). IPCW create a weighted population in which observed individuals are weighted to represent individuals who were lost to follow-up at 3 and 12 months based on observed characteristics. These characteristics included the same set of baseline confounders described above and additionally included paternal education and any IPV at baseline. IPCW were multiplied with sampling weights and used in generalized linear models. We used Stata 14,^46^ R (4.1.0),^47^ and RStudio^48^ for all analyses.

## Results

### Descriptive statistics

We present unweighted descriptive statistics on our analytic sample (n = 783) and weighted statistics (means, standard deviations (SDs), and percentages) using sampling weights to provide information on the baseline characteristics of our population-representative sample (Table 1). On average, mothers were 27 years old, 30% were having their first pregnancy, and 36% completed less than middle school (up to 8 years). Only 13% of mothers lived in nuclear families, 69% lived with a grandmother of the index child, and the average household size was 9.

**Table 1.** Sample characteristics, Bachpan cohort, Pakistan (n = 783).

	Unweighted		Weighted^a^	
	N/mean	%/SD	Mean	%/SD
Maternal characteristics				
Age (range: 18–45)	26.7	4.3	26.6	4.2
Education (years)				
None (0)	110	14.1	–	13.0
Primary (1–5) to middle (6–8)	300	38.3	–	36.2
Secondary or higher (9+)	373	47.6	–	50.8
Number of living children				
First pregnancy	221	28.2	–	29.6
1–3 children	500	63.9	–	63.5
4+ children	62	7.9	–	6.9
Past-year intimate partner violence				
No	409	52.2	–	56.1
Yes	374	47.8	–	43.9
Household characteristics				
Paternal education (years)				
None (0)	60	7.7	–	7.1
Primary (1–5) to middle (6–8)	242	30.9	–	30.0
Secondary or higher (9+)	481	61.4	–	62.9
Grandmother co-residence	538	68.7	–	69.0
Household size (range: 0–48)	8.5	4.7	8.5	4.8
People per room (range: 0–25)	2.4	1.8	2.3	1.8
Nuclear family	104	13.3	–	12.6
Socioeconomic status asset index (range: –5.0 to 2.8)	0.0	1.6	0.2	1.6
Trial arm				
Control	191	24.4	–	16.5
Intervention	198	25.3	–	17.1
Non-depressed	394	50.3	–	66.4
Index child gender				
Male	385	49.2	–	50.1
Female	398	50.8	–	49.9

SD: standard deviation.

a Means, SD, and percentages were weighted by sampling weights.

Table 2 presents unweighted and weighted descriptive statistics on MIL childcare, perinatal depression, and family conflict across the perinatal period. Given these variables were likely affected by loss to follow-up and to provide statistics on our population-representative sample, we used the final weight, which combined sampling weights and IPCW. After applying these weights, at 3 months, roughly 45% of MIL participated in 1–5 caregiving activities (low involvement) and 18% participated in 6 or more activities (high involvement). At 12 months, 28% of MIL had low involvement and 25% had high involvement. The most commonly reported activities were caring for the child when the mother was occupied with other tasks/chores (3 months: 52%; 12 months: 43%), unwell (3 months: 42%; 12 months: 41%), or working (3 months: 42%; 12 months: 42%); and MIL playing/interacting with the child (3 months: 39%; 12 months: 39%). The changes in MIL involvement at 3 versus 12 months were driven by specific activities. Over time, MIL became less involved in giving baths to the child and were more involved in feeding, providing snacks, holding, and soothing the child.

**Table 2.** Descriptive statistics on mother-in-law childcare and perinatal depression, Bachpan cohort, Pakistan, n = 783.

	Unweighted N (%)				Weighted (%)^a^		
	3 months		12 months		3 months	12 months	
Overall mother-in-law childcare							
No involvement (0)	304 (38.8)		369 (47.1)		(37.2)	(46.6)	
Low (1–5)	343 (43.8)		230 (29.4)		(44.6)	(28.3)	
High (6+)	136 (17.4)		184 (23.5)		(18.2)	(25.2)	
Instrumental care							
Sleep/naps	109 (13.9)		117 (14.9)		(13.8)	(14.9)	
Feeding meals	25 (3.2)		77 (9.8)		(3.2)	(9.8)	
Providing snacks to child	52 (6.6)		175 (22.4)		(6.6)	(22.3)	
Changing diapers/clothes or washing/cleaning	99 (12.6)		102 (13.0)		(12.6)	(13.0)	
Giving bath	165 (21.1)		88 (11.2)		(21.0)	(11.2)	
Non-instrumental care							
Playing/interacting with child	309 (39.5)		304 (38.8)		(39.2)	(38.7)	
Child likes to be held by the MIL the most	95 (12.1)		159 (20.3)		(12.1)	(20.3)	
Soothing child when restless/crying	127 (16.2)		176 (22.5)		(16.2)	(22.3)	
Other support (care for the child)							
When mother is occupied with other tasks/chores	405 (51.7)		341 (43.6)		(51.3)	(43.4)	
When mother is unwell	331 (42.3)		325 (41.5)		(41.9)	(41.3)	
When mother is working	329 (42.0)		328 (41.9)		(41.6)	(41.7)	
	Unweighted N (%)				Weighted (%)^a^		
	Baseline	3 months		12 months	Baseline	3 months	12 months
Perinatal depression	291 (37.2)	120 (15.3)		151 (19.3)	(26.0)	(13.2)	(16.1)
Any family conflict	314 (40.1)	–		186 (23.8)	(37.1)	–	(22.5)
Quarrels with other family members	153 (19.5)	–		109 (13.9)	(17.7)	–	(13.3)
Troubled relations with close relatives/friends	196 (25.0)	–		97 (12.4)	(22.9)	–	(11.9)
Marital problems	204 (26.1)	–		91 (11.6)	(23.4)	–	(10.3)

MIL: mothers-in-law.

a Percentages, means, and standard deviations were calculated using data weighted by sampling and censoring weights at the relevant timepoint.

The prevalence of perinatal depression dropped between pregnancy and 3 months, but increased at 12 months (baseline: 26%; 3 months: 13%; 12 months: 16%). The percentage of mothers reporting family conflict decreased between baseline and 12 months postpartum (37% vs 23%). At baseline, the most common conflict reported was marital problems (23%). At 12 months, this became the least common (10%), and quarrels with other family members became the most commonly reported conflict (13%). We now highlight our results examining the associations between MIL childcare and perinatal depression. Detailed effect estimates and precision measures are presented in appendices (Appendix Tables 2–3).

### Three-month postpartum

**Figure 1.**
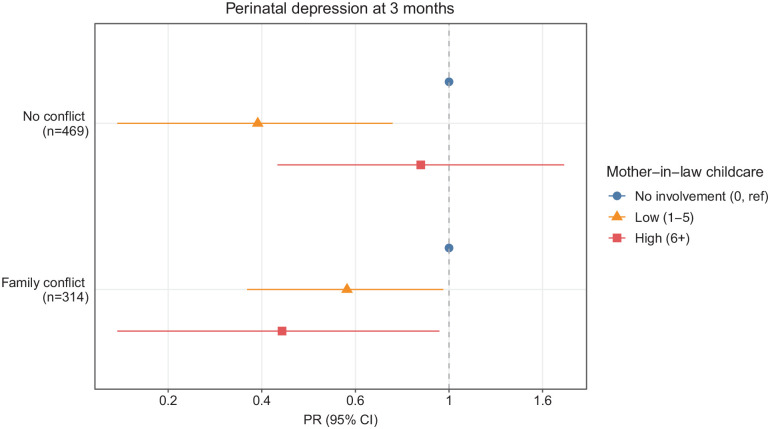
Cross-sectional associations between mother-in-law childcare and perinatal depression at 3 months, stratified by family conflict, Pakistan, n = 783. Three-month models were stratified by baseline family conflict and adjusted for baseline depression. PR: prevalence ratio; CI: confidence interval.

The relationship between MIL childcare and perinatal depression at 3 months varied by family conflict (Figure 1). Among households with no family conflict, 3-month MIL involvement in childcare was associated with lower perinatal depression at 3 months (Figure 1). Compared to no involvement, low and high MIL childcare involvement was associated with 0.36 [95% CI: 0.17, 0.74] and 0.86 [0.40, 1.85] times the prevalence of depression, respectively.

Among families with conflict, 3-month MIL childcare was associated with lower perinatal depression at 3 months postpartum (Figure 1). In particular, low levels of MIL childcare (vs no involvement) were associated with a lower prevalence of maternal depression (PR (95% CI): 0.58 (0.34, 0.97)). Moreover, high MIL childcare (vs no involvement) was associated with a reduced prevalence of depression (0.41 (0.17, 0.95)).

### Twelve-month postpartum

**Figure 2.**
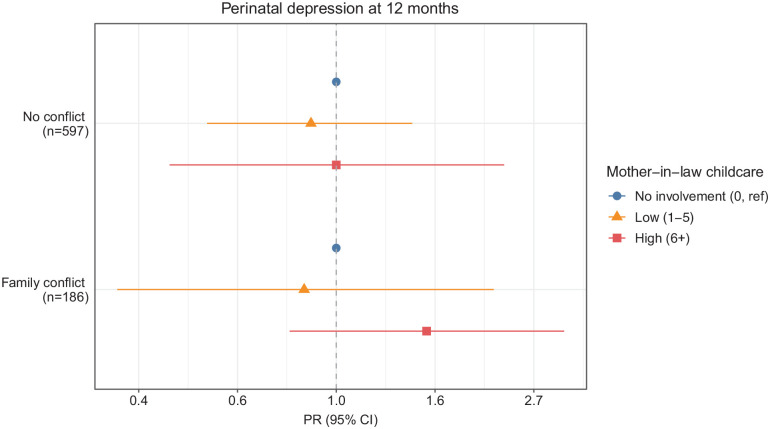
Cross-sectional associations between mother-in-law childcare and perinatal depression at 12 months, stratified by family conflict, Pakistan, n = 783. Twelve-month models were stratified by 12-month family conflict and adjusted for baseline and 3-month perinatal depression and 3-month mother-in-law childcare. PR: prevalence ratio; CI: confidence interval.

Similar to 3-month results, the relationship between MIL childcare on perinatal mental health at 12 months varied by family conflict (Figure 2). However, the precision of our estimates was low, as seen in the wide CIs (range of confidence limit ratios: 2.83–6.73). Among families with no conflict, we found no associations between 12-month MIL involvement in childcare and perinatal depression (Figure 2). However, among families with conflict, high MIL childcare (vs no involvement) was associated with a higher prevalence of perinatal depression (1.58 (0.79, 3.17)).

## Discussion

In our sample of rural Pakistani families, the majority of mothers reported MIL participating in daily childcare activities in the first year postpartum. MIL often provided substitute caregiving when mothers were occupied with other tasks, busy, or unwell. MIL were also commonly reported playing/interacting with and soothing their grandchild. We found cross-sectional associations between MIL involvement in childcare and perinatal depression, and these associations varied by family conflict.

MIL childcare had strong associations with perinatal depression in the immediate postpartum period. Low levels of 3-month MIL childcare, compared to no MIL involvement, were associated with lower perinatal depression among families with no conflict. Among families with conflict, both low and high levels of MIL childcare had benefits, with high levels associated with reductions in perinatal depression. Our results corroborate existing research demonstrating the benefits of increased traditional cultural practices and social support during the immediate postpartum period on maternal mental health.^5,6,9,10^ Further work is necessary to tease out the type and timing of MIL support to mothers in the first year after birth. In particular, understanding how MIL and DIL engage in child-rearing decision-making and the extent to which mothers find MIL childcare support beneficial to themselves (as opposed to the child) via qualitative methods may help to inform maternal mental health interventions.

Compared to 3 months postpartum, mothers reported a greater percentage of MIL involvement in childcare at 12 months, but this did not translate to improvements in the prevalence of perinatal depression. In particular, among families with no conflict, 12-month MIL childcare was not associated with perinatal depression. Yet, among families with conflict, high MIL childcare was associated with a greater prevalence of perinatal depression. One potential explanation for the attenuation and reversal of estimates is that while there are more childcare activities for MIL to participate in at 12 months versus 3 months, greater MIL involvement may lead to more tension between MIL and DIL as DIL become more confident and less stressed and MIL and DIL need to navigate caretaking responsibilities and child-rearing practices. For DIL, it may be that MIL involvement at 3 months is perceived as helpful because the immediate postpartum period is stressful, while at 12 months, MIL involvement may be perceived as interfering with the DIL’s way of child-rearing. For instance, we saw noticeable increases in MIL feeding/providing snacks as well as holding/soothing the child between 3 and 12 months. Increased MIL involvement in these activities may come with more opportunities for conflict, as MIL and DIL negotiate what, when, and how much to feed, or when and how to soothe the child. This, in turn, may lead to a dilution of the maternal mental health benefits of MIL childcare support. Furthermore, in stressful settings where mothers experience family conflict, greater MIL involvement may exacerbate these issues and increase the risk for perinatal depression. These results underscore the importance of contextualizing the family setting when studying social support and its relationship with perinatal depression. Indeed, our findings suggest that increasing MIL support outside of the initial postpartum period without addressing family conflict may not benefit maternal mental health.

In addition to the amount of childcare support, the timing and context of the support matter for perinatal depression. In the immediate postpartum period, MIL childcare support was linked to lower perinatal depression. During this time, high levels of MIL support were beneficial for mothers living in families with conflict, but not as much for mothers living in families with no conflict. It may be that mothers living in stressful environments during the immediate postpartum period require a higher level of support. At the end of the postpartum period, MIL childcare support was no longer beneficial, and in fact, for mothers living in environments of conflict between family members, high MIL involvement in childcare was linked to a greater prevalence of perinatal depression than if there was no MIL support at all. Our findings emphasize the importance of intervening early in the postpartum period to provide support to mothers and reduce perinatal depression risk. Moreover, the results highlight how stressful environments play an important role in moderating the relationship between social support and perinatal depression.

In addition, the source of social support is also critical for maternal mental health. In general, family and community members often view grandmothers as knowledgeable and experienced, and they often serve as key advisors to younger women and/or decision-makers on pregnancy and child caregiving issues.^49,50^ Yet, the distinction between paternal and maternal grandmother childcare support can have important and direct impacts on mothers. While we were unable to conduct such a comparison due to low reports of maternal grandmother childcare support (3 months: 13%; 12 months: 10%), prior work in urban China found that women who received traditional postpartum support (*zuo yuezi*) from their MIL had twice the odds of postpartum depression compared to those who received support from their own mothers.^51^ The dynamic responsibilities of child-rearing in the first year of life is a stressful time for mothers. Given their strong influence on younger women’s healthcare access and use,^25,52–54^ engaging MIL to provide high-quality support to mothers in the perinatal period may help prevent adverse mental health. Promising work from India demonstrates how counseling MIL led to higher exclusive breastfeeding rates compared to counseling husbands and even mothers themselves.^55^ While social support interventions may help mothers and children, careful consideration of who, what, when, and in what context the support is given is necessary to improve perinatal depression.

### Limitations and strengths

Our study has several limitations to discuss. First, a key limitation is that our measure of MIL involvement in childcare was maternally reported. Mothers may not have accurately or comprehensively reported MIL involvement, and this may differ by maternal depression and/or family conflict, potentially leading to misclassification bias. Future work should incorporate observation or MIL-reported information. A second limitation is the cross-sectional nature of our analyses. We conducted cross-sectional analyses because the childcare provided at 3 and 12 months postpartum are qualitatively distinct and because we hypothesized the impact of childcare support on depression is likely more immediate than long-term. We assumed MIL childcare precedes perinatal depression; however, perinatal depression can also affect the extent to which MIL step in as caregivers. We controlled for outcomes at prior waves to mitigate confounding by maternal history of depression, but we acknowledge the time ordering of MIL childcare and perinatal depression cannot be established. Future longitudinal work is necessary and should consider more frequent data collection across the perinatal period. Third, our measure of depression was SCID, a dichotomous indicator of clinical levels of depression. Symptom severity may provide a more nuanced understanding of the role of MIL childcare on depression. Sensitivity analyses using PHQ-9 at 3 months (not available at 12 months) showed similar results to our findings with SCID at 3 months. Fourth, selection bias due to loss to follow-up may bias our results. However, we implemented stabilized IPCW to account for informative loss to follow-up based on observed characteristics. We assumed any missing data aside from missing outcomes were missing completely at random. The only covariate that had missing data was any IPV (n = 42), which we used in our IPCW models. Given missing data on IPV is likely missing not at random (i.e. the value of the missing IPV is related to the reason why it is missing), we did not impute these data. However, we conducted sensitivity analyses in which we reran IPCW and outcome models twice, assuming all 42 missing observations (1) experienced IPV and (2) did not experience IPV. Results (not shown) were not meaningfully different. Finally, we did not have data on the age or health status of MIL, which may directly affect involvement in childcare activities and indirectly affect perinatal depression through increased caretaking responsibilities. Further work is necessary to understand whether and how MIL age and morbidity affect maternal mental health.

Despite these limitations, our study has multiple strengths. First, we extend the literature on family support and its role in perinatal depression by examining an understudied, yet influential family member: MIL. Second, we utilized detailed data on MIL childcare activities in the first year postpartum. The Day-in-the-Life measure provides a more comprehensive picture of MIL childcare than general social support to mothers during the perinatal period. Third, we used data from a population-representative sample of depressed and non-depressed women in rural Pakistan. Fourth, we used a validated measure of perinatal depression that assessed clinical levels rather than instruments that screen for depression. Finally, we explored how MIL childcare affected perinatal depression at two distinct time points in the postpartum period and how this relationship varied across contexts of family conflict.

## Conclusion

In summary, we found MIL childcare was associated with perinatal depression, and this varied by timing in the postpartum period and across contexts of family conflict. MIL childcare in the immediate postpartum period was especially important for maternal mental health. Our findings highlight the complexities of MIL/DIL relationship in the perinatal period for perinatal depression as well as the importance of family context when studying social support. Given the influential decision-making power of MIL and the communal nature of caregiving across many cultures, maternal mental health and child caregiving interventions should take a family-centered approach. Incorporating MIL and other family members into such interventions with careful consideration of family conflicts may help to improve intrahousehold knowledge, practices, and dynamics that can maximize benefits across generations to help mothers and children.

## Supplemental Material

sj-docx-1-whe-10.1177_17455057221141288 – Supplemental material for Mother-in-law childcare and perinatal depression in rural Pakistan Click here for additional data file. Supplemental material, sj-docx-1-whe-10.1177_17455057221141288 for Mother-in-law childcare and perinatal depression in rural Pakistan by Esther O Chung, Ashley Hagaman, Amina Bibi, Allison Frost, Sarah C Haight, Siham Sikander and Joanna Maselko in Women’s Health

## Declarations

**Ethics approval and consent to participate:** The Bachpan Cohort study received ethical approval from the institutional review boards at the Human Development Research Foundation (IRB/1017/2021), Duke University, and the University of North Carolina at Chapel Hill (#20-1433). Written informed consent, or witnessed informed consent if the participant was illiterate, was obtained before study participation.

**Consent for publication:** Not applicable.

**Availability of data and materials:** Requests for data can be made to Joanna Maselko, senior author.

## References

[1] Lasater ME Beebe M Gresh A , et al. Addressing the unmet need for maternal mental health services in low-and middle-income countries: integrating mental health into maternal health care. J Midwifery Womens Health 2017; 62(6): 657–660. 29149521 10.1111/jmwh.12679 PMC6696925

[2] Atif N Lovell K Rahman A. Maternal mental health: the missing “m” in the global maternal and child health agenda. Semin Perinatol 2015; 39(5): 345–352. 26164538 10.1053/j.semperi.2015.06.007

[3] Burger M Hoosain M Einspieler C , et al. Maternal perinatal mental health and infant and toddler neurodevelopment-evidence from low and middle-income countries. J Affect Disord 2020; 268: 158–172. 32174474 10.1016/j.jad.2020.03.023

[4] Śliwerski A Kossakowska K Jarecka K , et al. The effect of maternal depression on infant attachment: a systematic review. Int J Environ Res Public Health 2020; 17(8): 2675. 10.3390/ijerph17082675 PMC7216154 32295106

[5] LeMasters K Andrabi N Zalla L , et al. Maternal depression in rural Pakistan: the protective associations with cultural postpartum practices. BMC Public Health 2020; 20(1): 68. 31941468 10.1186/s12889-020-8176-0 PMC6964000

[6] Hagaman A LeMasters K Zivich PN , et al. Longitudinal effects of perinatal social support on maternal depression: a marginal structural modelling approach. J Epidemiol Community Health 2021; 75: 936–943. 33712512 10.1136/jech-2020-215836 PMC8434957

[7] Maselko J Bates L Bhalotra S , et al. Socioeconomic status indicators and common mental disorders: evidence from a study of prenatal depression in Pakistan. SSM Popul Health 2018; 4: 1–9. 29349268 10.1016/j.ssmph.2017.10.004 PMC5769091

[8] Maselko J Hagaman AK Bates LM , et al. Father involvement in the first year of life: associations with maternal mental health and child development outcomes in rural Pakistan. Soc Sci Med 2019; 237: 112421. 31398510 10.1016/j.socscimed.2019.112421 PMC6708722

[9] Chien L Tai C Ko Y , et al. Adherence to “Doing-the-month” practices is associated with fewer physical and depressive symptoms among postpartum women in Taiwan. Res Nurs Health 2006; 29(5): 374–383. 16977638 10.1002/nur.20154

[10] Grigoriadis S Cindylee D Kenneth F , et al. Postpartum cultural practices: a systematic review of the evidence. Ann Gen Psychiatry 2008; 7: S163.

[11] Zhong QY Gelaye B VanderWeele TJ , et al. Causal model of the association of social support with antepartum depression: a marginal structural modeling approach. Am J Epidemiol 2018; 187(9): 1871–1879. 29617921 10.1093/aje/kwy067 PMC6118064

[12] Li Y Long Z Cao D , et al. Social support and depression across the perinatal period: a longitudinal study. J Clin Nurs 2017; 26(17–18): 2776–2783. 28334472 10.1111/jocn.13817

[13] Kazi A Fatmi Z Hatcher J , et al. Social environment and depression among pregnant women in urban areas of Pakistan: importance of social relations. Soc Sci Med 2006; 63(6): 1466–1476. 16797813 10.1016/j.socscimed.2006.05.019

[14] Waite LJ Luo Y Lewin AC. Marital happiness and marital stability: consequences for psychological well-being. Soc Sci Res 2009; 38(1): 201–212.

[15] Cheng ER Rifas-Shiman SL Perkins ME , et al. The influence of antenatal partner support on pregnancy outcomes. J Womens Health 2016; 25(7): 672–679. 10.1089/jwh.2015.5462 PMC4985003 26828630

[16] Lin WC Chang SY Chen YT , et al. Postnatal paternal involvement and maternal emotional disturbances: the effect of maternal employment status. J Affect Disord 2017; 219: 9–16. 28501681 10.1016/j.jad.2017.05.010

[17] Drysdale RE Slemming W Makusha T , et al. Father involvement, maternal depression and child nutritional outcomes in Soweto, South Africa. Matern Child Nutr 2021; 17(Suppl. 1): e13177. 34241955 10.1111/mcn.13177 PMC8269140

[18] Kasamatsu H Tsuchida A Matsumura K , et al. Paternal childcare at 6 months and risk of maternal psychological distress at 1 year after delivery: the Japan environment and children’s study (JECS). Eur Psychiatry 2021; 64(1): e38. 34106043 10.1192/j.eurpsy.2021.2213 PMC8260565

[19] Allendorf K. Like her own: ideals and experiences of the mother-in-law/daughter-in-law relationship. J Fam Issues 2006; 55(5): 588–600. 27147777 10.1111/j.1741-3729.2006.00428.x PMC4852487

[20] Chung EO Hagaman A LeMasters K , et al. The contribution of grandmother involvement to child growth and development: an observational study in rural Pakistan. BMJ Glob Health 2020; 5(8): e2181. 10.1136/bmjgh-2019-002181 PMC7418670 32784209

[21] Bennett L. Dangerous wives and sacred sisters: Social and symbolic roles of high-caste women in Nepal. Religious Studies 1985; 21(2): 246–250.

[22] Jeffery P Jeffery R Lyon A. Labour pains and labour power: women and childbearing in India. London: Zed Books, 1989.

[23] Minturn L Kapoor S. Sita’s daughters: coming out of purdah: the Rajput women of Khalapur revisited Volume 10. Oxford: Oxford University Press, 1993.

[24] Rowther AA Kazi AK Nazir H , et al. “A woman is a puppet.” women’s disempowerment and prenatal anxiety in Pakistan: a qualitative study of sources, mitigators, and coping strategies for anxiety in pregnancy. Int J Environ Res Public Health 2020; 17(14): 4926. 32650551 10.3390/ijerph17144926 PMC7400614

[25] Mumtaz Z Salway SM. Gender, pregnancy and the uptake of antenatal care services in Pakistan. Sociol Health Illn 2007; 29(1): 1–26. 17286703 10.1111/j.1467-9566.2007.00519.x

[26] Gausia K Fisher C Ali M , et al. Antenatal depression and suicidal ideation among rural Bangladeshi women: a community-based study. Arch Womens Ment Health 2009; 12(5): 351–358. 19468825 10.1007/s00737-009-0080-7

[27] Chandran M Tharyan P Muliyil J , et al. Post-partum depression in a cohort of women from a rural area of Tamil Nadu, India: incidence and risk factors. Br J Psychiatry 2002; 181: 499–504. 12456520 10.1192/bjp.181.6.499

[28] Lee DT Yip AS Leung TY , et al. Ethnoepidemiology of postnatal depression: prospective multivariate study of sociocultural risk factors in a Chinese population in Hong Kong. Br J Psychiatry 2004; 184: 34–40. 14702225 10.1192/bjp.184.1.34

[29] Pearson V Phillips MR He F , et al. Attempted suicide among young rural women in the People’s Republic of China: possibilities for prevention. Suicide Life Threat Behav 2002; 32(4): 359–369. 12501961 10.1521/suli.32.4.359.22345

[30] Zaman RM. Parenting in Pakistan: an overview. In: Selin H (ed.) Parenting across cultures: childrearing, motherhood and fatherhood in non-western cultures. Dordrecht: Springer Netherlands, 2014, pp. 91–104.

[31] Turner EL Sikander S Bangash O , et al. The effectiveness of the peer-delivered Thinking Healthy PLUS (THPP+) program for maternal depression and child socioemotional development in Pakistan: study protocol for a randomized controlled trial. Trials 2016; 17(1): 442. 27608926 10.1186/s13063-016-1530-y PMC5017048

[32] Sikander S Ahmad I Atif N , et al. Delivering the thinking healthy programme for perinatal depression through volunteer peers: a cluster randomised controlled trial in Pakistan. Lancet Psychiatry 2019; 6(2): 128–139. 30686386 10.1016/S2215-0366(18)30467-X

[33] Maselko J Sikander S Turner EL , et al. Effectiveness of a peer-delivered, psychosocial intervention on maternal depression and child development at 3 years postnatal: a cluster randomised trial in Pakistan. Lancet Psychiatry 2020; 7(9): 775–787. 32828167 10.1016/S2215-0366(20)30258-3 PMC8015797

[34] Gallis JA Maselko J O’Donnell K , et al. Criterion-related validity and reliability of the Urdu version of the patient health questionnaire in a sample of community-based pregnant women in Pakistan. PeerJ 2018; 6: e5185. 30038858 10.7717/peerj.5185 PMC6054083

[35] O’Donnell KJ Gallis JA Turner EL , et al. The day-in-the-life method for assessing infant caregiving in rural Pakistan. Fam Relat. Epub ahead of print 3 June 2022. DOI: 10.1111/fare.12706. PMC10281745 37346745

[36] First MB Gibbon M . The structured clinical interview for DSM-IV axis I disorders (SCID-I) and the structured clinical interview for DSM-IV axis II disorders (SCID-II). In: Hilsenroth MJ Segal DL (eds.) Comprehensive handbook of psychological assessment, Vol 2: personality assessment. Hoboken, NJ: John Wiley & Sons, Inc., 2004, pp. 134–143.

[37] Gorman LL O’Hara MW Figueiredo B , et al. Adaptation of the structured clinical interview for DSM-IV disorders for assessing depression in women during pregnancy and post-partum across countries and cultures. Br J Psychiatry Suppl 2004; 46: s17–s23. 14754814 10.1192/bjp.184.46.s17

[38] Nast I Bolten M Meinlschmidt G , et al. How to measure prenatal stress? A systematic review of psychometric instruments to assess psychosocial stress during pregnancy. Paediatr Perinat Epidemiol 2013; 27(4): 313–322. 23772932 10.1111/ppe.12051

[39] Rahman A Iqbal Z Waheed W , et al. Translation and cultural adaptation of health questionnaires. J Pak Med Assoc 2003; 53(4): 142–147. 12776898

[40] Brown GW Harris TO. Life events and illness. London: The Guildford Press,1989.

[41] Husain N Creed F Tomenson B. Depression and social stress in Pakistan. Psychol Med 2000; 30(2): 395–402. 10824659 10.1017/s0033291700001707

[42] Rahman A Iqbal Z Harrington R. Life events, social support and depression in childbirth: perspectives from a rural community in the developing world. Psychol Med 2003; 33(7): 1161–1167. 14580070 10.1017/s0033291703008286

[43] Kolenikov S Angeles G. Socioeconomic status measurement with discrete proxy variables: is principal component analysis a reliable answer? Rev Income Wealth 2009; 55(1): 128–165.

[44] World Health Organization. WHO multi-country study on women’s health and domestic violence against women: initial results on prevalence, health outcomes and women’s responses. Geneva: World Health Organization, 2005.

[45] Zou G. A modified Poisson regression approach to prospective studies with binary data. Am J Epidemiol 2004; 159(7): 702–706. 15033648 10.1093/aje/kwh090

[46] StataCorp. Stata statistical software: release 14. College Station, TX: StataCorp LP, 2015.

[47] R Core Team. R: a language and environment for statistical computing. Vienna: R Foundation for Statistical Computing, 2021, https://www.R-project.org/

[48] RStudio Team. RStudio: integrated development environment for R. Boston, MA: RStudio, PBC, 2021, http://www.rstudio.com/

[49] Aubel J. The role and influence of grandmothers on child nutrition: culturally designated advisors and caregivers. Matern Child Nutr 2012; 8(1): 19–35. 21951995 10.1111/j.1740-8709.2011.00333.x PMC6860857

[50] Aubel J. Grandmothers: a learning institution. USAID, 2005, http://www.beps.net/publications/grandmothersfinaltag.pdf

[51] Wan EY Moyer CA Harlow SD , et al. Postpartum depression and traditional postpartum care in China: role of Zuoyuezi. Int J Gynaecol Obstet 2009; 104(3): 209–213. 19036364 10.1016/j.ijgo.2008.10.016

[52] White D Dynes M Rubardt M , et al. The influence of intrafamilial power on maternal health care in Mali: perspectives of women, men and mothers-in-law. Int Perspect Sex Reprod Health 2013; 39(2): 58–68. 23895882 10.1363/3905813

[53] Speizer IS Story WT Singh K. Factors associated with institutional delivery in Ghana: the role of decision-making autonomy and community norms. BMC Pregnancy Childbirth 2014; 14(1): 398. 25427853 10.1186/s12884-014-0398-7 PMC4247879

[54] Ganle JK Obeng B Segbefia AY , et al. How intra-familial decision-making affects women’s access to, and use of maternal healthcare services in Ghana: a qualitative study. BMC Pregnancy Childbirth 2015; 15(1): 173. 26276165 10.1186/s12884-015-0590-4 PMC4537557

[55] Smittenaar P Ramesh BM Jain M , et al. Bringing greater precision to interactions between community health workers and households to improve maternal and newborn health outcomes in India. Global Health Sci Pract 2020; 8(3): 358–371. 10.9745/GHSP-D-20-00027 PMC7541124 33008853

