# Supplementary material for: Mother-in-law childcare and perinatal depression in rural Pakistan
Source: Womens Health (Lond). 2022 Dec 5;18:17455057221141288. doi: 10.1177/17455057221141288 (PMC9726850; doi:10.1177/17455057221141288)
Supplement: sj-docx-1-whe-10.1177_17455057221141288 – Supplemental material for Mother-in-law childcare and perinatal depression in rural Pakistan [file sj-docx-1-whe-10.1177_17455057221141288.docx]

| **Appendix Table 1. A Day in Life (DiL) Questionnaire** | |
| --- | --- |
| Maternal grandmother (MGM); Paternal grandmother (PGM); Maternal grandfather (MGF); Paternal grandfather (PGF) | |
| **I. Instrumental care** | |
| For sleep routine please note who does each of the three following: | |
| 1. Morning waking | 1--- Mother only  2--- Mother and someone else together or taking turns  3--- Others mostly or others only  4--- No one, baby is alone/does on his/her own |
| 1a. Who else does it or helps?  (multiple options allowed) | 1--- MGM 2--- PGM 3--- MGF  4--- PGF 5--- SIBLING 6--- Aunt/Uncle  7--- Paid Help 8--- Father 9---Other___ |
| 2. Daytime nap(s) | 1--- Mother only  2--- Mother and someone else together or taking turns  3--- Others mostly or others only  4--- No one, baby is alone/does on his/her own |
| 2a. Who else does it or helps?  (multiple options allowed) | 1--- MGM 2--- PGM 3--- MGF  4--- PGF 5--- SIBLING 6--- Aunt/Uncle  7--- Paid Help 8--- Father 9---Other___ |
| 3. Sleeping at night | 1--- Mother only  2--- Mother and someone else together or taking turns  3--- Others mostly or others only  4--- No one, baby is alone/does on his/her own |
| 3a. Who else does it or helps?  (multiple options allowed) | 1--- MGM 2--- PGM 3--- MGF  4--- PGF 5--- SIBLING 6--- Aunt/Uncle  7--- Paid Help 8--- Father 9---Other___ |
| For meal time, please note who helps feed the child or supports mother in doing so. | |
| 4. Morning feed | 1--- Mother only  2--- Mother and someone else together or taking turns  3--- Others mostly or others only  4--- No one, baby is alone/does on his/her own |
| 4a. Who else does it or helps?  (multiple options allowed) | 1--- MGM 2--- PGM 3--- MGF  4--- PGF 5--- SIBLING 6--- Aunt/Uncle  7--- Paid Help 8--- Father 9---Other___ |
| 4o. Morning other feed. | 1--- Mother only  2--- Mother and someone else together or taking turns  3--- Others mostly or others only  4--- No one, baby is alone/does on his/her own |
| 4o(a). Who else does it or helps?  (multiple options allowed) | 1--- MGM 2--- PGM 3--- MGF  4--- PGF 5--- SIBLING 6--- Aunt/Uncle  7--- Paid Help 8--- Father 9---Other___ |
| 5. Afternoon meal | 1--- Mother only  2--- Mother and someone else together or taking turns  3--- Others mostly or others only  4--- No one, baby is alone/does on his/her own |
| 5a. Who else does it or helps?  (multiple options allowed) | 1--- MGM 2--- PGM 3--- MGF  4--- PGF 5--- SIBLING 6--- Aunt/Uncle  7--- Paid Help 8--- Father 9---Other___ |
| 6. Afternoon other food | 1--- Mother only  2--- Mother and someone else together or taking turns  3--- Others mostly or others only  4--- No one, baby is alone/does on his/her own |
| 6a. Who else does it or helps?  (multiple options allowed) | 1--- MGM 2--- PGM 3--- MGF  4--- PGF 5--- SIBLING 6--- Aunt/Uncle  7--- Paid Help 8--- Father 9---Other___ |
| 7. Evening feed | 1--- Mother only  2--- Mother and someone else together or taking turns  3--- Others mostly or others only  4--- No one, baby is alone/does on his/her own |
| 7a. Who else does it or helps?  (multiple options allowed) | 1--- MGM 2--- PGM 3--- MGF  4--- PGF 5--- SIBLING 6--- Aunt/Uncle  7--- Paid Help 8--- Father 9---Other___ |
| 8. Evening other food | 1--- Mother only  2--- Mother and someone else together or taking turns  3--- Others mostly or others only  4--- No one, baby is alone/does on his/her own |
| 8a. Who else does it or helps?  (multiple options allowed) | 1--- MGM 2--- PGM 3--- MGF  4--- PGF 5--- SIBLING 6--- Aunt/Uncle  7--- Paid Help 8--- Father 9---Other___ |
| **Other tasks** | |
| 9.Change diaper, clothes, washing during morning | 1--- Mother only  2--- Mother and someone else together or taking turns  3--- Others mostly or others only  4--- No one, baby is alone/does on his/her own |
| 9a. Who else does it or helps?  (multiple options allowed) | 1--- MGM 2--- PGM 3--- MGF  4--- PGF 5--- SIBLING 6--- Aunt/Uncle  7--- Paid Help 8--- Father 9---Other___ |
| 10. Change diaper, clothes, washing during afternoon | 1--- Mother only  2--- Mother and someone else together or taking turns  3--- Others mostly or others only  4--- No one, baby is alone/does on his/her own |
| 10a. Who else does it or helps?  (multiple options allowed) | 1--- MGM 2--- PGM 3--- MGF  4--- PGF 5--- SIBLING 6--- Aunt/Uncle  7--- Paid Help 8--- Father 9---Other___ |
| 11. Change diaper, clothes, washing during evening | 1--- Mother only  2--- Mother and someone else together or taking turns  3--- Others mostly or others only  4--- No one, baby is alone/does on his/her own |
| 11a. Who else does it or helps?  (multiple options allowed) | 1--- MGM 2--- PGM 3--- MGF  4--- PGF 5--- SIBLING 6--- Aunt/Uncle  7--- Paid Help 8--- Father 9---Other___ |
| 12. When mother is occupied with other tasks (chores which are regarding the house/herself, napping etc), in the morning the baby is with whom? | 1--- Mother only  2--- Mother and someone else together or taking turns  3--- Others mostly or others only  4--- No one, baby is alone/does on his/her own |
| 12a. Who else does it or helps?  (multiple options allowed) | 1--- MGM 2--- PGM 3--- MGF  4--- PGF 5--- SIBLING 6--- Aunt/Uncle  7--- Paid Help 8--- Father 9---Other___ |
| 13. When mother is occupied with other tasks (chores which are regarding the house/herself, napping etc.) in the afternoon, the baby is with whom? | 1--- Mother only  2--- Mother and someone else together or taking turns  3--- Others mostly or others only  4--- No one, baby is alone/does on his/her own |
| 13a. Who else does it or helps?  (multiple options allowed) | 1--- MGM 2--- PGM 3--- MGF  4--- PGF 5--- SIBLING 6--- Aunt/Uncle  7--- Paid Help 8--- Father 9---Other___ |
| 14. When mother is occupied with other tasks (chores which are regarding the house/herself, napping etc.) in the evening, the baby is with whom? | 1--- Mother only  2--- Mother and someone else together or taking turns  3--- Others mostly or others only  4--- No one, baby is alone/does on his/her own |
| 14a. Who else does it or helps?  (multiple options allowed) | 1--- MGM 2--- PGM 3--- MGF  4--- PGF 5--- SIBLING 6--- Aunt/Uncle  7--- Paid Help 8--- Father 9---Other___ |
| 15. Giving bath to the child | 1--- Mother only  2--- Mother and someone else together or taking turns  3--- Others mostly or others only  4--- No one, baby is alone/does on his/her own |
| 15a. Who else does it or helps?  (multiple options allowed) | 1--- MGM 2--- PGM 3--- MGF  4--- PGF 5--- SIBLING 6--- Aunt/Uncle  7--- Paid Help 8--- Father 9---Other___ |
| 16. Playing or interacting with child | 1--- Mother only  2--- Mother and someone else together or taking turns  3--- Others mostly or others only  4--- No one, baby is alone/does on his/her own |
| 16a. Who else does it or helps?  (multiple options allowed) | 1--- MGM 2--- PGM 3--- MGF  4--- PGF 5--- SIBLING 6--- Aunt/Uncle  7--- Paid Help 8--- Father 9---Other___ |
| 17. Who does the child like to be held by the most? | 1--- Mother only  2--- Mother and someone else together or taking turns  3--- Others mostly or others only  4--- No one, baby is alone/does on his/her own |
| 17a. Who else does it or helps?  (multiple options allowed) | 1--- MGM 2--- PGM 3--- MGF  4--- PGF 5--- SIBLING 6--- Aunt/Uncle  7--- Paid Help 8--- Father 9---Other___ |
| 18. Soothing the child when he/ she is restless/crying | 1--- Mother only  2--- Mother and someone else together or taking turns  3--- Others mostly or others only  4--- No one, baby is alone/does on his/her own |
| 18a. Who else does it or helps?  (multiple options allowed) | 1--- MGM 2--- PGM 3--- MGF  4--- PGF 5--- SIBLING 6--- Aunt/Uncle  7--- Paid Help 8--- Father 9---Other___ |
| 19. Care for the child when the mother is unwell | 1--- Mother only  2--- Mother and someone else together or taking turns  3--- Others mostly or others only  4--- No one, baby is alone/does on his/her own |
| 19a. Who else does it or helps?  (multiple options allowed) | 1--- MGM 2--- PGM 3--- MGF  4--- PGF 5--- SIBLING 6--- Aunt/Uncle  7--- Paid Help 8--- Father 9---Other___ |
| 20.Care for the child when the mother is working | 1--- Mother only  2--- Mother and someone else together or taking turns  3--- Others mostly or others only  4--- No one, baby is alone/does on his/her own |
| 20a. Who else does it or helps?  (multiple options allowed) | 1--- MGM 2--- PGM 3--- MGF  4--- PGF 5--- SIBLING 6--- Aunt/Uncle  7--- Paid Help 8--- Father 9---Other___ |

| **Appendix Table 2. The associations between mother-in-law childcare and perinatal depression at 3 months postpartum stratified by family conflict, Bachpan Cohort, Pakistan, n=783** | | | |
| --- | --- | --- | --- |
|  | **No Family Conflict (n=469)** | | |
|  | PR | 95% CI | CLR |
| MIL childcare (3mo) |  |  |  |
| Non-involved | *ref* | *ref* | *ref* |
| Low | 0.36 | (0.17, 0.74) | 4.35 |
| High | 0.86 | (0.40, 1.85) | 4.63 |
|  | **Family Conflict (n=314)** | | |
| MIL childcare (3mo) |  |  |  |
| Non-involved | *ref* | *ref* | *ref* |
| Low | 0.58 | (0.34, 0.97) | 2.85 |
| High | 0.41 | (0.17, 0.95) | 5.59 |
| All models used cluster robust standard errors and were conducted on weighted data to account for uneven sampling by baseline depression status and missingness at 3 and 12 months postpartum.  All models controlled for the following baseline covariates: child gender, maternal age, maternal education, number of living children (first pregnancy, 1-3, 4+), grandmother co-residence, household size, nuclear family, SES assets, and trial arm.  Three-month models were stratified by baseline family conflict and adjusted for baseline depression.  Abbreviations: Mother-in-law (MIL); Prevalence Ratio (PR); Confidence Interval (CI); Confidence Limit Ratio (CLR) | | | |
|  |  |  |  |
|  |  |  |  |
|  |  |  |  |

| **Appendix Table 3. The associations between mother-in-law childcare and perinatal depression at 12 months postpartum stratified by family conflict, Bachpan Cohort, Pakistan, n=783** | | | |
| --- | --- | --- | --- |
|  | **No Family Conflict (n=597)** | | |
|  | **PR** | **95% CI** | **CLR** |
| MIL childcare (12mo) |  |  |  |
| Non-involved | *ref* | *ref* | *ref* |
| Low | 0.88 | **(0.52, 1.47)** | **2.83** |
| High | 1.00 | **(0.43, 2.34)** | **5.44** |
|  | **Family Conflict (n=186)** | | |
| MIL childcare (12mo) |  |  |  |
| Non-involved | *ref* | *ref* | *ref* |
| Low | **0.85** | **(0.33, 2.22)** | **6.73** |
| High | **1.58** | **(0.79, 3.17)** | **4.01** |
| All models used cluster robust standard errors and were conducted on weighted data to account for uneven sampling by baseline depression status and missingness at 3 and 12 months postpartum.  All models controlled for the following baseline covariates: child gender, maternal age, maternal education, number of living children (first pregnancy, 1-3, 4+), grandmother co-residence, household size, nuclear family, SES assets, and trial arm.  Twelve-month models were stratified by 12-month family conflict, adjusted baseline and 3-month depression, and controlled for 3-month MIL childcare.  Abbreviations: Mother-in-law (MIL); Prevalence Ratio (PR); Confidence Interval (CI); Confidence Limit Ratio (CLR) | | | |
|  |  |  |  |
|  |  |  |  |
|  |  |  |  |
